# Continuous ketone monitoring in inborn errors of metabolism

**DOI:** 10.1016/j.metop.2026.100517

**Published:** 2026-10-28

**Authors:** Jaka Sikonja, Andrej Janez, Nadan Gregoric, Tadej Battelino, Urh Groselj

**Affiliations:** a Faculty of Medicine, University of Ljubljana, Vrazov Trg 2, 1000, Ljubljana, Slovenia; b Department of Endocrinology, Diabetes, and Metabolic Diseases, Division of Internal Medicine, University Medical Centre Ljubljana, Zaloska Cesta 2, 1000, Ljubljana, Slovenia; c Department of Endocrinology, Diabetes, and Metabolism, University Children's Hospital, University Medical Centre Ljubljana, Bohoriceva 20, 1000, Ljubljana, Slovenia

**Keywords:** Continuous ketone monitoring, CKM, Beta-hydroxybutyrate, Inborn errors of metabolism, Ketogenic diet

## Abstract

Disruptions in ketone metabolism can markedly impair energy homeostasis and clinical outcomes in individuals with certain inborn errors of metabolism (IEMs), making ketone monitoring an important component of disease management. Evidence for ketone monitoring has largely emerged from diabetes care, where it reduces the risk of diabetic ketoacidosis (DKA), hospitalizations, and healthcare costs. However, conventional ketone self-monitoring methods are limited by impracticality and lack of longitudinal data. Continuous ketone monitoring (CKM), which measures subcutaneous beta-hydroxybutyrate (BHB), overcomes these limitations by enabling real-time assessment of ketone dynamics. In this narrative review, we discuss emerging CKM technologies for the first time and outline their potential clinical utility in IEMs. However, direct clinical evidence supporting the use of CKM in IEMs is currently lacking, and further studies are needed to validate these proposed applications. In theory, CKM could improve metabolic control, seizure management, and prevent complications in disorders of ketolysis and in individuals with IEMs on ketogenic dietary therapies; help in early detection of catabolic states and metabolic decompensation; and enhance surveillance for DKA in IEM-associated insulin-deficient diabetes. Given the predictable metabolic vulnerability of patients with IEMs and the need for timely intervention, these disorders may represent a clinically meaningful early application of CKM beyond diabetes.

## Highlights

• CKM could enable real-time assessment of ketonemia in inborn errors of metabolism.
• In theory, CKM could also be used in individuals with inborn errors of metabolism.
• Further studies are needed to determine whether CKM improves clinical outcomes.

## Abbreviations

BHB –: beta-hydroxybutyrate
CGM –: continuous glucose monitoring
CKM –: continuous ketone monitoring
DGK –: dual glucose-ketone
DKA –: diabetic ketoacidosis
Glut1 –: glucose transporter type 1
Glut1DS –: glucose transporter type 1 deficiency syndrome
GSD –: glycogen storage disease
IEM –: inborn error of metabolism
KD –: ketogenic diet
PDC –: pyruvate dehydrogenase complex
PDCD –: pyruvate dehydrogenase complex deficiency
SCOT – succinyl-CoA: 3-oxoacid CoA transferase
SCOTD – succinyl-CoA: 3-oxoacid CoA transferase deficiency
T2 –: mitochondrial acetoacetyl-CoA thiolase
T2 deficiency –: mitochondrial acetoacetyl-CoA thiolase deficiency

## 1 Introduction

Ketosis is a physiological metabolic state in response to limited carbohydrate availability. The main ketone bodies (or ketones) are acetoacetate, beta-hydroxybutyrate (BHB) and acetone, and are produced in the liver from fatty acids to provide an alternative source of energy-rich molecules for high-demand organs including the brain, myocardium, and skeletal muscle [1]. Ketone metabolism encompasses both ketogenesis, the hepatic process by which fatty acids are converted into ketone bodies, and ketolysis, which involves the utilization of these ketone bodies by extrahepatic tissues to generate energy [2]. Physiological ketosis occurs during fasting, prolonged starvation, intense exercise, the neonatal period, pregnancy, and adherence to low-carbohydrate ketogenic diets (KDs) [3]. Ketone metabolism is tightly regulated by hormones: insulin inhibits ketogenesis and promotes lipogenesis, whereas glucagon and catecholamines stimulate both lipolysis and ketogenesis [1].

In healthy individuals, circulating BHB is typically <0.5 mmol/L in the fed state, rises to ∼0.5–5.0 mmol/L during nutritional/therapeutic ketosis (e.g., KDs, short-to-moderate fasting), and may reach up to 10 mmol/L with prolonged fasting, while systemic pH remains normal due to residual insulin activity, renal ketone handling, and buffering capacity [4], [5]. In contrast, ketoacidosis reflects a maladaptive state in which ketone production outpaces utilization and buffering, leading to high ketonemia and metabolic acidemia. In diabetes, this most commonly occurs in the setting of absolute or relative insulin deficiency with excess counterregulatory hormones, producing the triad of hyperglycemia, ketonemia, and acidosis, characteristic of diabetic ketoacidosis (DKA) [6], [7]. Contemporary DKA criteria include hyperglycemia (blood glucose >11 mmol/L), ketonemia (BHB ≥3.0 mmol/L or urine ketones ≥2+), and metabolic acidosis (pH < 7.3 or bicarbonate <18 mmol/L) to define DKA [8]. A general overview of different ketotic states is presented in Table 1.

**Table 1:** Differences between ketotic states with quantitative ranges for beta-hydroxybutyrate (BHB).

State	Typical BHB (mmol/L)	Acid–base status	Clinical rationale
Physiological fasting/short-term carbohydrate restriction	∼0.5–3.0	Normal	Adaptive
Therapeutic ketogenic diet	∼0.5–5.0 (individualized targets)	Normal	Intended/desired
Prolonged fasting (days)	up to ∼8–10	Usually normal (acidemia in severe cases)	Adaptive (catabolic)
Ketoacidosis (e.g., DKA)	≥3.0 (often much higher)	Acidemia	Life-threatening

Ketone metabolism has attracted considerable interest in the study of inborn errors of metabolism (IEMs), as alterations in ketogenesis and ketolysis can significantly impact energy homeostasis and clinical outcomes in these disorders [9]. Ketoacidosis typically occurs in IEMs due to defects in enzymes essential for ketolysis [2]. In certain IEMs, therapeutic ketosis is introduced by KD, aiming to restore energy utilization or alleviate disease-related symptoms [10]. These clinical situations require regular ketone monitoring to achieve optimal outcomes. Until recently, ketone monitoring was impractical, poorly accessible, and infrequently used; however, the advent of continuous ketone monitoring (CKM) systems may offer new possibilities in the management of patients with IEMs.

Evidence supporting ketone monitoring has largely emerged from diabetes management, where DKA is a relatively common and life-threatening complication. This narrative review extends this perspective to IEMs, focusing on the emerging applications of novel CKM systems in this patient population, with these potential applications being primarily theoretical at present due to the absence of direct clinical evidence in individuals with IEMs.

## 2 Methods

This narrative review was conducted to evaluate the hypothetical feasibility, potential clinical utility, and applicability of CKM systems for the management of individuals with selected IEMs. A comprehensive literature search of the PubMed database was performed on 9 January 2026. No restrictions on publication date were applied. Only articles published in English were included, and all types of publications were considered. Two advanced search queries were used: the first combined ketone-related terms with IEMs, while the second specifically focused on CKM. Detailed search strategies are described in the Supplementary Material. Study selection was performed independently by two researchers. Titles and abstracts were first screened for relevance, followed by full-text assessment of potentially eligible articles. Articles were included based on their relevance to ketone metabolism and the clinical applications of ketone monitoring in IEMs. Given the limited evidence on the use of CKM outside diabetes, we focused on IEMs in which ketone measurement could be used to guide clinical care. We summarized the existing evidence on intermittent ketone monitoring and discussed the potential clinical applications of CKM in these disorders based on this evidence, while clearly acknowledging the current limitations and evidence gaps. To support this conceptual framework, we also incorporated evidence from continuous glucose monitoring (CGM) as a related technology and provided relevant background on the underlying IEMs to facilitate interpretation of the findings. Given the narrative nature of the review, no formal risk-of-bias assessment was conducted.

## 3 Ketone measurement

In people with diabetes, frequent ketone monitoring combined with an appropriate home-based emergency management plan can help prevent the onset of DKA; identify individuals at higher risk for future DKA; reduce the need for hospitalization or emergency department visits, and contribute to lower healthcare costs [11], [12]. Unfortunately, adherence to ketone monitoring during episodes of hyperglycemia and acute illness is low despite clear recommendations [13]. Similarly, proactive ketone monitoring may reduce the risk of ketoacidotic episodes in individuals with disorders of ketolysis and serve as a biomarker of adherence to KD [5].

Point-of-care measurement or self-measurement of ketones can be performed from urine, exhaled breath or capillary blood. Urine ketone test strips provide a semi-quantitative assessment of acetoacetate via its reaction with nitroprusside on the dipstick, resulting in a color change. This method does not detect BHB, is affected by certain medications, and is prone to result misinterpretation due to color discrimination errors [6]. Acetone in exhaled breath can be detected non-invasively using portable breath ketone analyzers; however, while more convenient than urine dipsticks and finger-prick blood testing, these devices are not widely adopted in clinical practice due to a lack of standardization and insufficient real-world clinical data [14]. Until recently, the preferred approach for self-monitoring of ketones has been the measurement of BHB in capillary blood using a ketone meter. This method offers several advantages over other approaches. Since BHB reaches the highest concentrations during DKA, methods that measure BHB provide a more reliable identification of both the onset and resolution of ketoacidosis compared with methods that assess other ketone bodies, such as acetoacetate via urine dipsticks or acetone in exhaled breath. During the onset of DKA, BHB is produced preferentially over acetoacetate; therefore, urine acetoacetate measurements may underestimate total ketone concentrations and the severity of ketoacidosis. Similarly, during treatment, BHB is oxidized to acetoacetate, leading to a decline in BHB levels and a paradoxical increase in urinary acetoacetate [6], [15]. Among individuals with type 1 diabetes, capillary blood ketone monitoring is preferred over urine ketone testing and is associated with lower rates of hospitalization for DKA [11].

For ketoacidotic states, such as DKA, measurement of BHB is preferred, as it provides the most clinically useful information to guide therapeutic decisions. Although point-of-care blood ketone meters are relatively inexpensive, widely accessible, and generally acceptable to patients, they still require a minimally invasive finger-prick to obtain capillary blood, necessitate a conscious decision to perform the measurement, and do not provide information on BHB trends or automatic alerts to notify the patient without requiring active attention [16].

## 4 Continuous glucose monitoring in inborn errors of metabolism

The advent of CGM systems has revolutionized the management of type 1 diabetes and, more recently, type 2 diabetes, largely replacing traditional self-monitoring of blood glucose via finger-prick testing [17]. The physical and psychological burden is a major factor limiting the integration of capillary blood self-monitoring into the daily lives of people with diabetes. In contrast to capillary blood self-monitoring, CGM systems are practical, improve glycemic control, reduce the frequency of hypoglycemic episodes, and provide additional information on short-term glucose variability and real-time glucose levels, with automatic alerts to guide patients in diabetes management.

Growing evidence suggests that CGM can be successfully applied beyond diabetology, including in metabolic syndrome, endocrine disorders [18], individuals with obesity without diabetes [19], pregnancy [20], nutritional and sports medicine [21], and in individuals with IEMs characterized by episodes of hypoglycemia [22].

Glycogen storage diseases (GSDs) are a group of IEMs characterized by defects in glycogen synthesis or breakdown and can cause hypoglycemic episodes during longer periods of fasting. A recent survey among GSD family members and healthcare professionals highlighted the benefits of CGM systems in GSD and supported their inclusion in the standard of care. CGM systems are perceived to provide more comprehensive insights into glucose levels and trends compared with traditional finger-prick testing. The greatest benefit lies in their ability to detect asymptomatic hypoglycemia, particularly since patients with GSD are typically younger at presentation than individuals with diabetes mellitus and may have difficulties communicating symptoms to their caregivers [23]. In patients with GSD, the use of CGM improved the detection of hypoglycemia and hyperglycemia and enabled individualized adjustment of dietary interventions, leading to a reduction in time spent in the hypoglycemic range, reduced glycemic variability, improved nocturnal glycemic stability, and reduced severity of hypoglycemic episodes, potentially reducing brain impairment in the long-term [22], [24]. Congenital hyperinsulinism is caused by dysregulated insulin secretion, resulting in recurrent hypoglycemia that can lead to neurocognitive impairment. In these patients, CGM systems have been shown to be superior to finger-prick glucose monitoring in detecting hypoglycemia, particularly nocturnal episodes, and to improve quality of life and the sense of safety for both patients and their caregivers [25], [26]. CGM systems have also been used for the detection of hypoglycemia in conditions involving defects in gluconeogenesis, including hereditary fructose intolerance and fructose-1,6-bisphosphatase deficiency [27], [28].

To conclude, the use of CGM has improved the management of patients with certain IEMs, and similar principles could conceptually be extended to CKM in selected IEMs, although their clinical applicability remains to be established.

## 5 Continuous ketone monitoring

CKM uses a ketone sensor to repeatedly measure BHB in subcutaneous tissue based on a BHB dehydrogenase enzymatic reaction [29]. Previous studies have demonstrated the feasibility of continuous ketone measurement using a spectroscopy-based approach in an animal model, as well as employing a microneedle device with an NAD-dependent dehydrogenase enzyme in both a skin-mimicking phantom gel model and artificial interstitial fluid [30], [31].

In 2021, Alva et al. demonstrated for the first time that a CKM sensor, with structure and dimensions comparable to those used in CGM, is feasible under both *in vitro* and *in vivo* conditions, requiring only a single factory calibration. For *in vitro* experiments, the sensor was characterized in a phosphate-buffered solution into which ketones were sequentially introduced. The sensor responded to changes in ketone concentration within 4 min and exhibited a strong positive linear correlation over the 0–8 mmol/L range. Its operational stability under these conditions was robust, with a total signal loss of only 2.1% over 14 days. The clinical component of the study included 12 participants adhering to a low-carbohydrate diet. Over the 14-day monitoring period, interstitial fluid BHB concentrations closely tracked capillary blood levels, with 82.4% of sensor readings falling within 0.225 mmol/L/20% and 91.4% within 0.3 mmol/L/30% of reference [29].

The measurement range of these CKM sensors (0–8 mmol/L) is comparable to that of commercially available blood ketone test strips and encompasses the ketone concentrations typically observed during monitoring of individuals with IEMs (Table 2) [29]. This provides a rational basis for exploring the potential application of CKM in IEMs; however, whether CKM can improve metabolic control, reduce the risk of metabolic decompensation and comorbidity development, enhance patients’ quality of life, and reduce mortality remains to be determined.

**Table 2:** Blood beta-hydroxybutyrate (BHB) levels reported in patients with different inborn errors of metabolism.

Condition	Reported blood BHB concentrations (mmol/L)	Reference
Succinyl-CoA:3-oxoacid CoA transferase deficiency	3.7–8.62 (during decompensation)	[32], [33], [34]
	0.4–0.7 (after overnight fasting)	
Mitochondrial acetoacetyl-CoA thiolase deficiency^a^	Increased (during decompensation)	[35], [36]
	Absent (during asymptomatic period)	
Treatment with ketogenic diet (in general)	0.5–5.0	[5]
Ketogenic diet for seizure control	2.77–5.6^b^	[37], [38]
Glucose transporter type 1 deficiency syndrome	2.0–5.0	[9]
Pyruvate dehydrogenase complex deficiency	3.0–3.5	[39], [40]
Treatment with ketogenic diet in glycogen storage disease type IIIa	0.5–4.0^c^	[41], [42]
Propionic and methylmalonic acidemia	Increased (during decompensation)	[43], [44]

a No specific reports of BHB in the blood; however, marked elevations of BHB and acetoacetate were reported in urine.

b Range calculated based on the 95% confidence intervals constructed from data reported in referenced studies.

c Approximate range derived from the figures presented in referenced studies.

## 6 Continuous dual glucose-ketone monitoring

In recent years, efforts to enable the simultaneous measurement of both glucose and ketones have expanded considerably. Initially, combined portable glucose–ketone meters that use separate test strips for each measurement came to the forefront; a more recent innovations in this field include continuous dual glucose–ketone (DGK) monitoring technologies (also referred to as CGM-CKM or CGKM sensors) capable of simultaneously measuring interstitial glucose and BHB using a single sensor [45].

A continuous DGK sensor, developed by Abbott Diabetes Care, uses adjacent glucose-sensing and ketone-sensing electrodes on a single sensor that is the same size as the FreeStyle Libre 3 and has *in vitro* characteristics similar to those of standalone CKM and CGM sensor with a response time of less than 5 min and a high correlation with buffer concentrations of both analytes. The sensor was shown to have little between-subject variation and is factory calibrated as user-based calibration may not be possible for a ketone sensor due to very low BHB concentrations under normal conditions [46].

Integrating the monitoring of both metabolites may improve adoption of the technology by eliminating the need for two separate devices, enhance glycemic control, and facilitate timely interventions to prevent ketoacidosis or maintain target ketosis. DGK sensors have the potential for use in pregnancy and diabetes management where they can be integrated within hybrid closed-loop systems [47], [48], but could also be used by a subset of patients with IEMs (in example GSD IIIa and IIIb) who require monitoring of glycemia and ketone concentrations for optimal outcomes [45].

## 7 Potential use of continuous ketone monitoring in inborn errors of metabolism

We identified four clinical contexts where CKM could potentially be applied to support symptom management and quality of life, prevent complications, and improve overall metabolic control in individuals with IEMs. These proposed applications draw on physiological rationale, current use of KD and monitoring of therapeutic ketosis, and recommendations for intermittent blood or urine ketone monitoring during metabolic stability, as part of home sick-day plans, and during metabolic decompensation. They also incorporate extrapolations from recent recommendations for CKM in diabetes management. Table 3 distinguishes the supporting evidence for each application.

• **Disorders of ketolysis:** CKM could support monitoring of metabolic control in patients with disorders of ketone utilization, such as succinyl-CoA:3-oxoacid CoA transferase deficiency (SCOTD) and mitochondrial acetoacetyl-CoA thiolase deficiency (T2 deficiency), by tracking deviations from an individual patient's usual ketone profile that may signal impending metabolic decompensation. By contrast, CKM is expected to have limited usefulness in ketogenesis disorders or fatty acid oxidation defects, due to absent or extremely low serum ketone levels.
• **Monitoring therapeutic ketosis:** In selected IEMs treated with a KD, CKM could provide real-time assessment of ketosis and an indirect measure of dietary adherence, complementing dietary records and intermittent ketone measurements. These data could guide timely dietary adjustments to address the underlying energy metabolism defects or control symptoms such as seizures. Reliable assessment of dietary adherence remains one of the major methodological limitations of nutritional studies, which is crucial in the management of numerous IEMs. Thus, CKM could support trials of ketogenic dietary therapy by providing continuous biochemical data on ketosis, interpreted alongside dietary records.
• **Indirect monitoring of catabolic states and metabolic decompensation:** An unexpected rise in BHB detected by CKM, interpreted alongside clinical signs and symptoms, could provide an early warning of metabolic stress at home and help identify impending metabolic decompensation in patients prone to deterioration during fasting or increased catabolism, and in patients with IEMs that affect ketone metabolism.
• **Monitoring for DKA in IEM-associated insulin-deficient diabetes:** By detecting rising ketone concentrations, CKM may help identify impending DKA, allowing prompt intervention to prevent severe metabolic complications. This proposed application is extrapolated from current evidence and recommendations for diabetes management in general.

**Table 3:** **Rationale and evidence supporting proposed CKM applications in inborn errors of metabolism (IEMs).** Note that none of these applications has been clinically validated using CKM in IEM populations. Beta-hydroxybutyrate (BHB) alone cannot establish dietary adherence, pathological catabolism, or ketoacidosis. Abbreviations: CKM – continuous ketone monitoring; DKA – diabetic ketoacidosis; Glut1DS – glucose transporter type 1 deficiency syndrome; GSD IIIa – glycogen storage disease type IIIa; KD – ketogenic diet; MMA – methylmalonic aciduria; PA – propionic acidemia; PDCD – pyruvate dehydrogenase complex deficiency; SCOT – succinyl-CoA:3-oxoacid CoA transferase; T2 – mitochondrial acetoacetyl-CoA thiolase.

Application	Physiological rationale	Supporting evidence	Reference
**Disorders of ketolysis**	Defective peripheral ketone utilization; rising BHB may signal impending ketoacidotic decompensation, particularly relative to the patient's baseline BHB.	Case reports and series of patients with SCOT or T2 deficiency, using intermittent ketone measurements; recommendations for home ketone testing during illness in these disorders.	[2], [36], [49], [50]
**Monitoring therapeutic ketosis**	Low carbohydrate intake reduces insulin levels, promoting lipolysis, fatty-acid β-oxidation, and ketogenesis; BHB reflects therapeutic ketosis during KD and may help assess dietary adherence and treatment response.	Intermittent ketone measurements in patients with Glut1DS, PDCD and GSD IIIa treated with KD, and in individuals with drug-resistant epilepsy of various etiologies; disease-specific monitoring recommendations.	[5], [9], [37], [38], [39], [40], [41], [42], [51]
**Indirect monitoring of catabolism and metabolic decompensation**	Catabolic states lower the insulin-to-counterregulatory hormone ratio, promoting lipolysis, fatty acid β-oxidation, and hepatic ketogenesis. In PA and MMA, accumulated toxic metabolites may further impair tricarboxylic acid cycle activity, favoring the diversion of acetyl-CoA toward ketone synthesis; an unexpected BHB rise, interpreted alongside clinical findings, may signal metabolic stress and warn of impending decompensation.	Intermittent ketone measurements during decompensation in patients with PA or MMA; recommendations for ketone testing during acute illness and metabolic decompensation in PA and MMA.	[43], [44], [52], [53]
**Monitoring for DKA in IEM-associated insulin-deficient diabetes**	Insulin deficiency promotes ketogenesis; serum BHB correlates with bicarbonate and pH; high diagnostic accuracy of BHB for DKA	Evidence from intermittent ketone testing and sick-day management in people with diabetes, primarily type 1 diabetes; CKM recommendations for diabetes populations extrapolated to IEM-associated insulin-deficient diabetes.	[11], [12], [54]

### 7.1 Disorders of ketolysis

Under physiological conditions, blood ketone bodies (BHB and acetoacetate) produced in the liver are transported via the circulation to extrahepatic tissues, where BHB is first converted to acetoacetate. Acetoacetate is then activated to acetoacetyl-CoA by succinyl-CoA:3-oxoacid CoA transferase (SCOT). In the final step, mitochondrial acetoacetyl-CoA thiolase (T2) cleaves acetoacetyl-CoA into two molecules of acetyl-CoA, which can be used for energy production [2]. Pathogenic variants in either of the two enzyme-encoding genes disrupt the ketolytic pathway.

#### 7.1.1 Succinyl-CoA:3-oxoacid CoA transferase deficiency

Succinyl-CoA:3-oxoacid CoA transferase deficiency (SCOTD; OMIM: 245050) is a rare autosomal recessive disorder of ketone body utilization and results from a genetic defect in *OXCT1*. To date, only around 40 patients with SCOTD have been reported in the literature, all of whom experienced characteristic acute ketoacidotic crises before the age of 3 years, although they may be completely asymptomatic between episodes. Most patients with SCOTD develop normally and do not experience organ-specific complications. Overall prognosis is favorable, provided that the disorder is diagnosed early and metabolic crises are promptly recognized and appropriately managed [49]. Acute decompensation is marked by potentially life-threatening severe metabolic acidosis, pronounced ketosis and ketonuria, and altered consciousness, whereas glucose levels are variable [49]. Apart from nonspecific elevations of both BHB and acetoacetate, no diagnostic metabolites are detectable in the blood or urine of these patients [55]. Persistent ketosis, even during asymptomatic anabolic states, is considered pathognomonic for SCOTD, although it is not present in around 43% of affected individuals [49]. During the onset of metabolic crisis, ketone levels in blood and urine increase, and some patients in the literature have been recommended to routinely check for ketonuria, especially during prolonged fasting, febrile episodes, or infections [32].

#### 7.1.2 Mitochondrial acetoacetyl-CoA thiolase deficiency

Mitochondrial acetoacetyl-CoA thiolase deficiency (T2 deficiency; OMIM: 203750) is caused by pathogenic variants in the *ACAT1* gene, which encodes T2, and results in impairment of both ketolysis and isoleucine catabolism [35]. During ketolysis, the step catalyzed by T2 can be partially compensated by the related mitochondrial medium-chain 3-ketoacyl-CoA thiolase in the liver. As a result, ketolysis is not completely abolished even in the complete absence of functional T2 enzyme activity [2]. A recent review of 135 cases reported that patients experience intermittent episodes of ketoacidosis, with initial presentations typically occurring between 6 and 36 months of age; however, some patients did not experience any metabolic decompensation [50]. Episodes of metabolic crisis are characterized by severe, potentially life-threatening metabolic acidosis, ketonuria, altered mental status, and variable blood glucose levels ranging from hypoglycemia to hyperglycemia [36]. Overall prognosis is generally favorable, but some patients develop neurological dysfunction, which is usually attributed to severe acidosis during metabolic crises; notably, neurological impairments can also occur in the absence of overt decompensation [50]. Effective management relies on preventing metabolic crises, which may be detected early using ketone monitoring.

### 7.2 Monitoring therapeutic ketosis for targeting energy homeostasis defect

#### 7.2.1 Glucose transporter type 1 deficiency syndrome

Glucose normally crosses the blood–brain barrier and astrocyte membrane via glucose transporter type 1 (Glut1). Mutations in the *SLC2A1* gene impair Glut1-mediated transport, reducing cerebral glucose availability and disrupting brain energy metabolism. Glucose transporter type 1 deficiency syndrome (Glut1DS; OMIM: 606777) is characterized by seizures, paroxysmal eye-head movements, developmental delay, movement disorders and intellectual disability. The metabolic hallmark of Glut1DS is low glucose concentration in the cerebrospinal fluid despite normal serum glucose levels [56]. In patients with Glut1DS, KD should be initiated as early as possible and maintained through adolescence and adulthood to provide ketones as an alternative cerebral energy source, thereby bypassing the transporter defect. KD has been shown to effectively control seizures and improve movement impairment and cognitive function [9]. The international Glut1DS study group recommends monitoring BHB levels in capillary or venous blood, targeting concentrations of 2–5 mmol/L, and considers qualitative urine ketone testing insufficient for monitoring ketosis during KD therapy [9].

#### 7.2.2 Pyruvate dehydrogenase complex deficiency

Pyruvate dehydrogenase complex deficiency (PDCD) is a mitochondrial disorder characterized by impaired conversion of pyruvate to acetyl-CoA, a critical step linking glycolysis to the tricarboxylic acid cycle. In PDCD, pathogenic variants in genes encoding subunits of the pyruvate dehydrogenase complex (PDC) lead to partial or complete loss of enzyme activity. The metabolic block leads to accumulation of pyruvate, which is shunted toward lactate, causing lactic acidosis. Reduced acetyl-CoA availability limits tricarboxylic acid cycle flux and oxidative phosphorylation, resulting in cellular energy failure, particularly in tissues with high energy demands such as the brain [39]. The dominant clinical features of the disorder include developmental delay, hypotonia, seizures, microcephaly and structural abnormalities of the brain, particularly cerebral atrophy, ventriculomegaly and dysgenesis of the corpus callosum [57]. Because fatty acid–derived ketone bodies can bypass the PDC and provide acetyl-CoA directly, ketones partially compensate for the metabolic defect. In these patients, KD counteracts the progressive neurodegeneration and has positive effects on seizures, sleep disturbances and motor and neurocognitive functioning. The effectiveness of the KD is primarily determined by the PDCD phenotype and by maintaining adequate ketosis. Regular monitoring of serum BHB levels is recommended to allow timely dietary adjustments and maintain target concentrations of 3.0–3.5 mmol/L for optimal outcomes [39], [40].

#### 7.2.3 Glycogen storage disease type IIIa

Glycogen storage disease type IIIa (GSD IIIa; OMIM 232400) is caused by deficiency of the glycogen debranching enzyme, encoded by the *AGL* gene, resulting in the accumulation of abnormally structured glycogen with short outer chains. Because glycogen plays a key role in maintaining serum glucose during fasting, these patients often experience hypoglycemic episodes. Progressive accumulation of abnormal glycogen in various tissues can cause hepatomegaly with elevated liver transaminases, muscle weakness or exercise intolerance, and, in some cases, hypertrophic cardiomyopathy [58]. Traditionally, management of hepatic GSDs has focused on a carbohydrate-rich diet with frequent daytime and nighttime feedings to prevent hypoglycemia. However, this approach can promote hyperinsulinemia and increase glycogen deposition, which is thought to play a central role in the pathogenesis of cardiac involvement in GSD IIIa [41]. More recently, high-fat or ketogenic diets have been reported to reduce cardiac hypertrophy, including interventricular septal thickness and left ventricular mass index, improve heart failure symptoms, and lower pro-B-type natriuretic peptide while minimizing hypoglycemia risk. Whether these cardiac improvements result from reduced intracellular glycogen accumulation, the provision of an alternative energy substrate, or both remains unclear [41], [42], [59], [60]. BHB monitoring has rarely been reported in patients with GSD IIIa receiving low-carbohydrate diets. Available data are limited to sparse intermittent measurements, with serum BHB concentrations ranging from 0.5 to 4.0 mmol/L; but whether these concentrations are associated with cardiac improvement remains unclear [41], [42].

### 7.3 Monitoring ketogenic diet adherence for symptom control

In both adults and children with medication-refractory epilepsy, seizure control can be significantly improved through the use of a KD [51]. Consequently, the KD has been applied with varying degrees of success in several IEMs – such as nonketotic hyperglycinemia, argininosuccinate lyase deficiency, and adenylosuccinate lyase deficiency – conditions in which epilepsy is a prominent clinical feature [10]. KD induces a metabolic shift in which ketone bodies replace glucose as the primary energy source for the brain. This shift is associated with reduced neuronal excitability, enhanced inhibitory neurotransmission, improved mitochondrial function, and modulation of neuroinflammatory pathways, all of which contribute to improved seizure control [61]. Cerebral ketone metabolism depends largely on circulating ketone body concentrations, particularly BHB, as well as on the efficiency of ketone transport across the blood–brain barrier. In a cohort of 74 children with refractory seizures of various etiologies on KD, serum BHB levels correlated well with seizure control and provided a more accurate assessment of ketosis compared to urine nitroprusside-based measurement of ketones [37]. Similarly, a study involving 33 individuals aged 1–40 years with therapy-resistant epilepsy demonstrated that a single capillary BHB measurement correlated more strongly with seizure reduction than either a single urine dipstick measurement or average ketonuria from multiple home urine samples [38]. Current guidelines recommend ketone monitoring using either urinary ketones or serum BHB; however, both methods reflect only single time-point measurements and do not provide information on trends over time. As KD should be maintained for a minimum of three months to adequately assess its therapeutic effect [51], CKM could provide a more comprehensive assessment of ketosis than urinary ketone testing or finger-prick BHB measurements, supporting evaluation of KD adherence and treatment effectiveness.

### 7.4 Monitoring ketogenic diet adherence in clinical trials

Dietary clinical trials inherently differ from pharmaceutical trials because diet represents a complex, multifaceted intervention rather than a single, standardized compound. Non-adherence and participant attrition can substantially reduce internal validity and bias estimates of dietary effects. Most nutritional studies continue to rely on self-reported dietary assessment tools, which lack objective verification and do not allow for continuous tracking of adherence. Although objective biomarkers could offer more reliable measures of dietary intake and adherence, their use remains limited due to cost, participant burden, and logistical constraints [62], [63]. On the other hand, the KD has the advantage that it induces nutritional ketosis, resulting in elevated ketone bodies that can be measured objectively—BHB in capillary blood or acetoacetate in urine [5]. Blood and urine ketone levels fluctuate throughout the day, following a circadian pattern. Therefore, the timing of sample collection is critical for accurately monitoring ketosis, with ketonuria being most reliably detected in the first morning urine and again later in the evening after dinner [64]. By providing continuous data, CKM could offer a more objective measure of adherence to the KD and facilitate the interpretation of outcomes in dietary clinical trials [5]. However, circulating BHB reflects both ketone production and utilization and is influenced by fasting, illness, exercise, and exogenous ketone intake; it therefore cannot independently establish adherence to a prescribed diet.

### 7.5 Ketone monitoring as a surrogate marker of catabolic status

In several IEMs, acute and potentially life-threatening metabolic crises can occur due to pathological catabolism, which may be triggered by infection, fever, trauma, medication, reduced food intake, or fasting. These metabolic crises can manifest as hypoglycemia, metabolic acidosis, hyperammonemia, lactic acidosis, encephalopathy, or multi-organ failure [65]. Physiological catabolism during fasting is a regulated adaptation: reduced insulin action promotes lipolysis, while hepatic fatty acid β-oxidation supplies acetyl-CoA for ketogenesis, increasing BHB as an alternative fuel. Feedback mechanisms restrain ketone production and preserve metabolic homeostasis [66]. During acute illness, counterregulatory hormones and inflammatory signals promote insulin resistance, gluconeogenesis, lipolysis, and protein breakdown. This initially adaptive response becomes pathological when excessive or sustained [67]. Increased fatty acid availability can enhance hepatic ketogenesis, which may be further amplified by metabolic blocks in selected IEMs, such as in propionic and methylmalonic acidemia. Stress-related catabolism does not invariably raise BHB, and concentrations can overlap substantially between physiological ketosis and pathological decompensation. Interpretation should therefore focus on an unexpected rise above the patient's usual metabolically stable BHB profile, alongside signs and symptoms of acute stress or impending decompensation. In this context, CKM could complement home sick-day plans by providing continuous BHB data as an indirect marker of catabolic activity, interpreted alongside clinical signs and symptoms. Unlike many disease-specific metabolites requiring laboratory testing, BHB could be monitored continuously at home, potentially benefiting patients prone to decompensation during fasting or metabolic stress, including those with selected organic acidurias.

#### 7.5.1 Organic acidurias

In propionic acidemia and methylmalonic acidemia, the enzymatic block leads to the accumulation of propionyl-CoA, methylmalonyl-CoA, and related toxic metabolites, which disrupt tricarboxylic acid cycle function and impair mitochondrial energy production. These disturbances may reduce acetyl-CoA utilization through the tricarboxylic acid cycle and favor its diversion toward ketogenesis. Consequently, ketosis and ketoacidosis have been reported in individuals with propionic acidemia and methylmalonic acidemia, particularly during periods of metabolic decompensation or catabolic stress [43], [44], [68], [69]. Acid–base status and plasma ammonia are key parameters for distinguishing metabolic decompensation from stability and assessing its severity in both disorders [52], [53]. In propionic acidemia, plasma ketone concentrations have also been positively correlated with plasma ammonia [43]. Monitoring of ketone levels is recommended during acute decompensations in propionic acidemia and methylmalonic acidemia [43], [44], [52]. Thus, detection of BHB via CKM could serve as a surrogate marker for impending metabolic decompensation, allowing home-based monitoring and timely implementation of interventions to improve clinical outcomes.

### 7.6 Monitoring for diabetic ketoacidosis

Endocrine disorders may arise as complications of various IEMs. The underlying pathophysiological mechanisms leading to endocrine gland dysfunction are multifactorial and primarily include acute or progressive intoxication resulting from the accumulation of normal or atypical metabolites upstream of the metabolic block; defects in cellular energy metabolism that impair normal endocrine function; and abnormalities in the metabolism of complex molecules, which can disrupt the structure and function of endocrine tissues [70]. Diabetes mellitus is one of the most common endocrinopathies observed in IEMs. It may result from impaired insulin secretion by pancreatic β-cells, which is usually not associated with an autoimmune etiology, or may be accompanied by liver- or muscle-derived insulin resistance [71]. Patients with substantial insulin deficiency are at particularly high risk of DKA. In this subgroup, similarly to type 1 diabetes, CKM may be beneficial for preventing DKA and reducing the need for hospitalization [70].

## 8 Ketone thresholds and actions

In the context of diabetes management, traffic-light displays of glucose concentrations have been implemented in CGM systems to facilitate reporting and alerting of users, caregivers, and healthcare professionals, and have recently been proposed for application in CKM systems [54], [72]. To support intuitive interpretation of continuous ketone data in IEMs, we propose two conceptual reporting frameworks, neither of which has been clinically validated in these populations. A *progressive traffic-light system* was adopted for CKM reporting in diabetes to reflect the escalating urgency of rising ketone concentrations and to prompt timely intervention [54]. A similar reporting system could be applied to those IEMs where, similarly to diabetes, low serum ketone concentrations are expected and desirable during a stable metabolic state and where a rise in ketone concentrations requires immediate attention to prevent metabolic decompensation (Fig. 1). On the other hand, nutritional ketosis is intentionally maintained with a KD in some IEMs, thus a *target-range-based traffic-light system* (Fig. 2), similar to a target glucose range system in diabetes, could be applied in this setting, as BHB concentrations within a target range are desired for optimal metabolic control. Although diabetes-oriented ketone thresholds informed the design of the proposed frameworks, their numerical boundaries cannot be assumed to have the same clinical significance across IEM subtypes, and the available evidence is insufficient to establish definitive CKM-derived BHB thresholds for these populations.

**Fig. 1:**
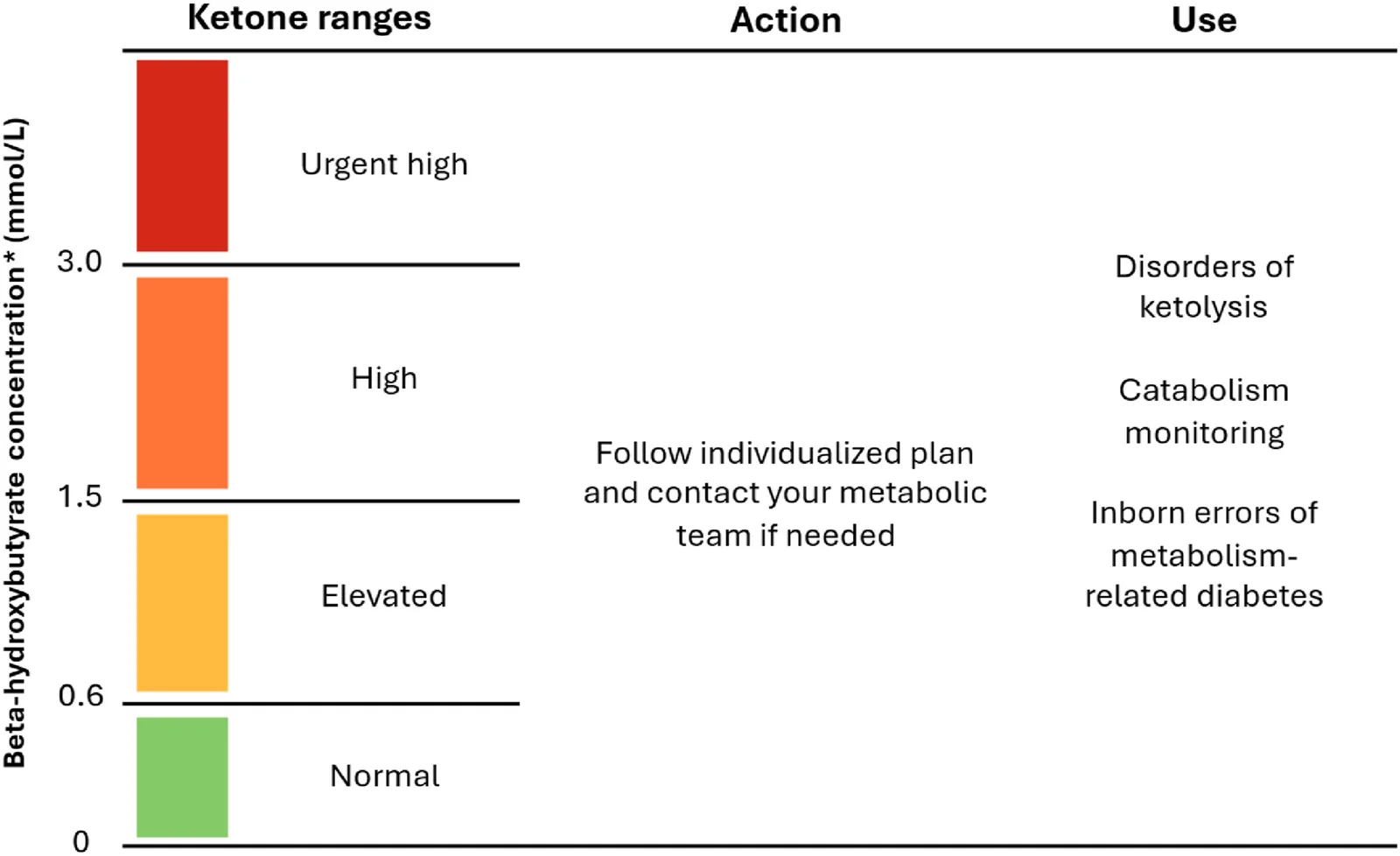
**Proposed and currently unvalidated progressive traffic-light system for interpretation of beta-hydroxybutyrate (BHB) concentrations, provided by continuous ketone monitoring, in individuals with inborn errors of metabolism (IEMs) not on a ketogenic diet and practical actions for system users.** A progressive traffic-light system is proposed, as rising BHB levels indicate increasing urgency. Thresholds established for diabetes could serve as an initial reference for this group; however, further studies are needed to define thresholds specific to individual IEMs and, potentially, individual patients. * Illustrative and unvalidated thresholds; future thresholds will require disease- and patient-specific validation.

**Fig. 2:**
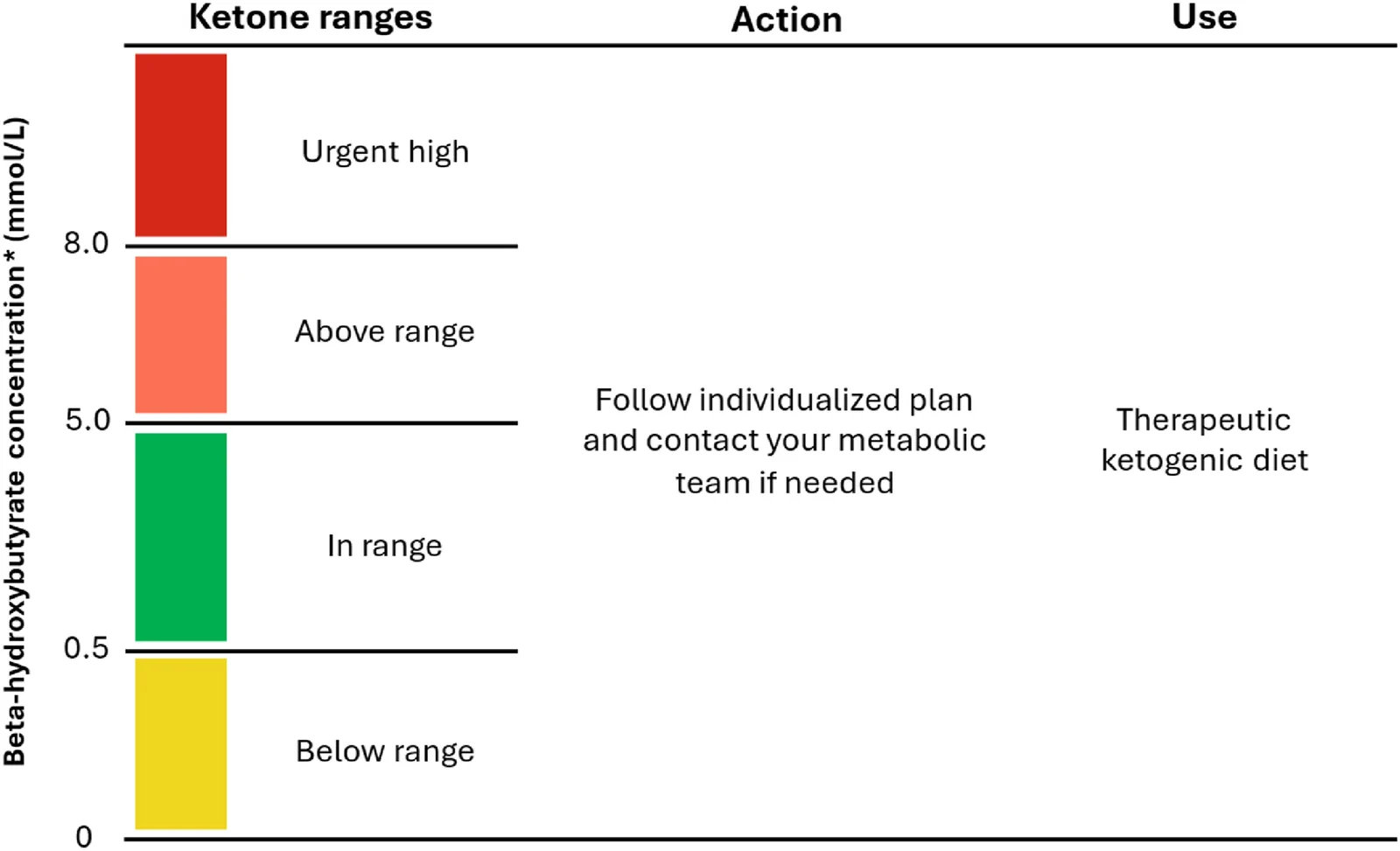
**Proposed and currently unvalidated target-range-based traffic-light system for interpretation of beta-hydroxybutyrate (BHB) concentrations, provided by continuous ketone monitoring, in individuals with inborn errors of metabolism (IEMs) treated with a ketogenic diet and practical actions for system users.** A target-range-based traffic-light system could be adopted for individuals with IEMs treated with a ketogenic diet, in whom nutritional ketosis is intentionally maintained. In this group of patients, BHB concentrations should be kept within a target range, analogous to glucose targets in diabetes management. A broad BHB target range is proposed based on current evidence from intermittent ketone monitoring across various disorders. Because ketogenic dietary therapy may also be associated with ketoacidosis, an “urgent high” ketone category was included with an arbitrary, unvalidated threshold. Precise thresholds cannot currently be established and will likely need to be tailored to specific IEMs and individual patients. * Illustrative and unvalidated thresholds; future thresholds will require disease- and patient-specific validation.

We hypothesize that CKM thresholds recommended for diabetes in general [54] may also be applicable to IEM-associated insulin-deficient diabetes and have therefore used the same BHB boundaries in Fig. 1 to illustrate the proposed framework. In ketolysis disorders and IEMs in which BHB may serve as an indirect marker of catabolism, intermittent blood ketone measurements indicate that BHB concentrations can rise during acute metabolic decompensation to levels comparable to those observed in DKA (Table 2). However, this overlap does not validate the application of diabetes-derived thresholds across IEM subtypes, and BHB concentrations alone are insufficient to diagnose ketoacidosis.

In Fig. 2, a broad BHB target range of 0.5–5.0 mmol/L was selected to reflect concentrations reported during ketogenic dietary therapy across various clinical conditions, including those measured by intermittent blood ketone monitoring in individuals with different IEMs, because a narrower range could not be defined based on the current evidence. An arbitrary upper alert threshold of 8.0 mmol/L was included to prompt clinical assessment for excessive ketosis and possible ketoacidosis during ketogenic dietary therapy or prolonged fasting [73], [74].

For both proposed frameworks, universal BHB thresholds may not be appropriate. Future studies should establish thresholds tailored to specific IEMs, taking into account age, disease burden, BHB concentrations at baseline and during metabolic decompensation, and tolerance of ketogenic dietary therapy. BHB concentrations should be interpreted alongside clinical symptoms and signs, and unexpected sensor readings should be verified by blood BHB measurements whenever feasible.

## 9 Current knowledge gaps

In this article, we summarized the currently available evidence supporting ketone monitoring across different clinical scenarios in IEMs; however, this evidence is derived from intermittent ketone monitoring, as direct evidence supporting the use of CKM in IEMs is currently lacking. Through our review, we identified several important gaps in the existing knowledge and key questions that should be addressed in future research:

− **Ketone monitoring in general:** As is common in rare disorders, the evidence supporting ketone monitoring—whether in urine or serum—is limited for most specific IEMs. Moreover, the marked heterogeneity of IEMs with respect to pathophysiology, age of onset, and metabolic risk limits the generalizability of ketone monitoring strategies and complicates the development of unified thresholds or clinical decision pathways.
− **Ketone monitoring using CKM systems:** Data supporting the use of CKM in IEMs are currently completely non-existent. Nevertheless, this review provides a theoretical framework in which CKM could potentially improve clinical outcomes in selected patient populations.
− **Clinically meaningful ketone ranges:** There is a substantial lack of data defining optimal or target ketone levels in IEMs. While some evidence exists for intermittent serum BHB measurements and urinary ketones, there is a complete absence of data establishing target ranges for CKM. In addition, there is no consensus on clinically meaningful ketone-related endpoints, further limiting study comparability and interpretation. The proposed traffic-light systems for ketone concentrations were developed as preliminary tools based on current theoretical knowledge; however, the proposed thresholds have not been clinically validated, and it remains uncertain whether their use translates into improved clinical outcomes.
− **Metabolic control:** It has yet to be established whether CKM provides a superior marker of metabolic control compared with traditional biochemical parameters and blood or urine ketones. Additionally, whether patients and caregivers can effectively use real-time CKM data to adjust dietary intake or medications in a timely manner and prevent complications and metabolic decompensations remains to be established.
− **Impact of CKM on clinical outcomes:** It remains unclear whether CKM can reduce the risk of metabolic decompensation and decrease the need for hospitalization or emergency visits.
− **Lag time between serum and interstitial BHB levels:** While the time delay between the actual glucose concentration in the blood and the glucose value measured by current CGM systems is a well-known limitation, it remains unclear whether a similar limitation also applies to CKM. In theory, BHB may diffuse faster than glucose, given that glucose has a higher molecular weight than BHB [46]. Consequently, lag time may have a less pronounced impact on CKM sensor measurements compared to CGM.
− **Acute states:** Uncertainties remain regarding the reliability of CKM in acute clinical settings characterized by rapid changes in serum ketone concentrations, the development of severe acidosis, peripheral hypoperfusion and subcutaneous tissue edema.
− **Interference with sensor measurements:** Analytical specificity should be evaluated against medications and metabolites that may accumulate in the intended IEM populations. Candidate interferents warranting investigation include branched-chain α-ketoacids, which characteristically accumulate in maple syrup urine disease, and 3-hydroxyisobutyric acid, a valine metabolite structurally similar to BHB [75]. However, neither a shared ketone functional group nor structural similarity alone establishes interference with an enzymatic BHB assay; BHB itself is a hydroxyacid rather than a ketone in the chemical sense. Interference from these metabolites therefore remains an untested possibility rather than an established sensor limitation. If cross-reactivity with 3-hydroxyisobutyric acid were demonstrated and analytically characterized, it could motivate investigation of sensor-based biochemical monitoring in the exceptionally rare disorder 3-hydroxyisobutyric aciduria (OMIM: 236795). Such an application would require clinical validation and a means of distinguishing the contributions of BHB and 3-hydroxyisobutyric acid to the sensor signal.
− **Other technical challenges:** Future studies should clarify whether CKM sensors ensure accuracy across the full BHB range in patients, require sensor warm-up periods, and whether factory calibration is sufficient.

## 10 Conclusions

In certain IEMs, CKM could theoretically offer new opportunities to support symptom management, prevent complications, and optimize metabolic control. Given the predictable metabolic vulnerabilities of these disorders and the substantial burden of conventional monitoring on patients and caregivers, IEMs may represent a potentially relevant area for future CKM application beyond diabetes. However, CKM is an emerging technology, and its proposed applications in IEMs remain hypothetical at this stage, as direct clinical evidence in this patient population is not yet available. Further studies are therefore needed to establish its feasibility, clinical utility, and potential role in the management of IEMs.

## CRediT authorship contribution statement

**Jaka Sikonja:** Writing – review & editing, Writing – original draft, Investigation, Conceptualization. **Andrej Janez:** Writing – review & editing, Writing – original draft, Validation, Investigation. **Nadan Gregoric:** Writing – review & editing, Writing – original draft, Investigation. **Tadej Battelino:** Writing – review & editing, Writing – original draft, Validation, Investigation, Funding acquisition. **Urh Groselj:** Writing – review & editing, Writing – original draft, Investigation, Conceptualization.

## Patient consent statement

Not applicable.

## Ethics considerations

Not applicable.

## Data sharing statement

Not applicable.

## Originality of content

We confirm all information and materials in the manuscript are original.

## Declaration of generative AI and AI-assisted technologies in the manuscript preparation process

During the preparation of this work, the authors used ChatGPT in order to improve the clarity and readability of the language used. After using this tool, the authors reviewed and edited the content as needed and take full responsibility for the content of the published article.

## Financial support

This work was supported by the Slovenian Research and Innovation Agency (grant: P3-0343). The funding organization had no role in the design and conduct of the study; collection, management, analysis, and interpretation of the data; preparation, review, or approval of the manuscript; and decision to submit the manuscript for publication.

## Declaration of competing interest

The authors declare the following financial interests/personal relationships which may be considered as potential competing interests: Urh Groselj reports article publishing charges was provided by Slovenian Research and Innovation Agency. Andrej Janez reports a relationship with Abbott that includes: consulting or advisory, speaking and lecture fees, and travel reimbursement. Tadej Battelino reports a relationship with Abbott that includes: board membership, consulting or advisory, funding grants, paid expert testimony, speaking and lecture fees, and travel reimbursement. Tadej Battelino reports a relationship with Slovenian Research and Innovation Agency that includes: funding grants. Tadej Battelino reports a relationship with National Institutes of Health that includes: funding grants. Tadej Battelino reports a relationship with Breakthrough T1D that includes: funding grants. Tadej Battelino reports a relationship with The Leona M and Harry B Helmsley Charitable Trust that includes: funding grants. Tadej Battelino reports a relationship with European Union that includes: funding grants. If there are other authors, they declare that they have no known competing financial interests or personal relationships that could have appeared to influence the work reported in this paper.
