# Supplementary material for: Continuous ketone monitoring in inborn errors of metabolism
Source: Metabol Open. 2026 Oct 28;32:100517. doi: 10.1016/j.metop.2026.100517 (PMC13744896; doi:10.1016/j.metop.2026.100517)
Supplement: Supplementary file 1 [file mmc1.docx]

**SUPPLEMENTARY MATERIAL**

Two advanced search queries were used in PubMed:

1. combining ketone-related terms with terms for inborn errors of metabolism (IEMs)

*("Ketone Bodies"[Mesh] OR ketones[Title/Abstract] OR "ketone bodies"[Title/Abstract] OR "beta-hydroxybutyrate"[Title/Abstract] OR "β-hydroxybutyrate"[Title/Abstract] OR "ketone monitoring"[Title/Abstract] OR "continuous ketone monitoring"[Title/Abstract] OR "ketogenic diet"[Mesh] OR "ketogenic diet"[Title/Abstract] ) AND ( "Inborn Errors of Metabolism"[Mesh] OR "inborn errors of metabolism"[Title/Abstract] OR "inherited metabolic disease*"[Title/Abstract] OR "inherited metabolic disorder*"[Title/Abstract])*

2. focused search on continuous ketone monitoring

Two advanced search queries were used for the search on pubmed: the first combined ketone-related terms with inborn errors of metabolism (IEMs), while the second specifically focused on continuous ketone monitoring.

*("continuous ketone monitoring" OR "continuous ketone monitor*" OR "continuous ketone sensor*" OR "continuous ketone measurement")*
